# Recent Advances in Our Understanding of the Diversity and Roles of Chaperone-Usher Fimbriae in Facilitating *Salmonella* Host and Tissue Tropism

**DOI:** 10.3389/fcimb.2020.628043

**Published:** 2021-02-03

**Authors:** Rachel A. Cheng, Martin Wiedmann

**Affiliations:** Department of Food Science, Cornell University, Ithaca, NY, United States

**Keywords:** *Salmonella*, fimbriae, chaperone-usher, host-pathogen interaction, adhesin *Salmonella*

## Abstract

*Salmonella enterica* is one of the most diverse and successful pathogens, representing a species with >2,600 serovars with a variety of adaptations that enable colonization and infection of a wide range of hosts. Fimbriae, thin hair-like projections that cover the surface of *Salmonella*, are thought to be the primary organelles that mediate *Salmonella*’s interaction with, and adherence to, the host intestinal epithelium, representing an important step in the infection process. The recent expansion in genome sequencing efforts has enabled the discovery of novel fimbriae, thereby providing new perspectives on fimbrial diversity and distribution among a broad number of serovars. In this review, we provide an updated overview of the evolutionary events that shaped the *Salmonella* chaperone-usher fimbriome in light of recent phylogenetic studies describing the population structure of *Salmonella enterica*. Furthermore, we discuss the complexities of the chaperone-usher fimbriae-mediated host-pathogen interactions and the apparent redundant roles of chaperone-usher fimbriae in host and tissue tropism.

## Introduction

The World Health Organization reported that *Salmonella* infections contributed the greatest burden of foodborne disease of any diarrheal disease agent, resulting in an estimated 8.6 million [95% confidence interval (CI) 3.9–17.5 million] disability-adjusted life years (DALYs) (Havelaar et al., 2015). *Salmonella* includes just two different species (*S. bongori* and *S. enterica*), but at least 2,659 known serovars (Issenhuth-Jeanjean et al., 2014). Among the six recognized subspecies of *S. enterica* (Brenner et al., 2000), the majority of serovars associated with human clinical disease belong to *S. enterica* subsp. *enterica* (Centers for Disease Control and Prevention, 2016). Serovars are often divided into typhoidal (serovar Typhi), paratyphoidal (serovars Paratyphi A, B, and C, and Sendai/Miami) and nontyphoidal serovars (e.g., serovars Typhimurium and Enteritidis), to reflect the diseases that they cause, with infection with typhoidal and paratyphoidal serovars resulting in an invasive, extraintestinal infection, and infection with nontyphoidal serovars resulting in a primarily gastrointestinal illness that is often self-limiting (Crump et al., 2015). An ongoing challenge in the effort to reduce the morbidity and mortality associated with human salmonellosis, is that many *Salmonella* serovars are known to colonize and infect a wide range of hosts, although some serovars are host-restricted (e.g., Typhi in humans) or host-adapted (e.g., Dublin in cows). Furthermore, potential reservoirs of many nontyphoidal serovars that are commonly associated with human clinical illness, remain largely unknown.

Reflective of its overall success at colonizing a large range of hosts to enhance its distribution throughout many environments, *Salmonella* uses a variety of strategies to survive passage through the host gastrointestinal tract, enabling its transient or long-term presence, and facilitating its spread to additional host populations. Fimbriae (also known as pili) are the thin, hair-like appendages that mediate bacterial adherence to a surface, such as the intestinal epithelium (Nuccio and Bäumler, 2007), although non-fimbrial adhesins have also been described (Wagner and Hensel, 2011). Given the importance of fimbriae in mediating host interactions for multiple pathogens (Kaper et al., 2004; Nobbs et al., 2009; Paczosa and Mecsas, 2016), special attention has been given to characterizing the role that different *Salmonella* fimbriae play in mediating host and tissue tropism, as well as their potential use in source attribution predictions (Zhang et al., 2019).

The recent increases in the number of *Salmonella* whole genome sequences (WGS) available has afforded the discovery and characterization of fimbriae encoded by a variety of *Salmonella* (Nuccio and Bäumler, 2007; Worley et al., 2018), as most fimbriae are poorly expressed under standard culturing conditions (Humphries et al., 2003; Thanassi et al., 2007; Hansmeier et al., 2017). In this review, we discuss recent advances in our understanding of the content and evolution of the *Salmonella* chaperone-usher fimbriome, and evidence supporting the complexity of fimbriae-mediated interactions in the context of tissue and host tropism.

## A Collector’s item: *Salmonella* Encodes a Diverse Number of Chaperone-Usher fimbriae

**Figure 1:**
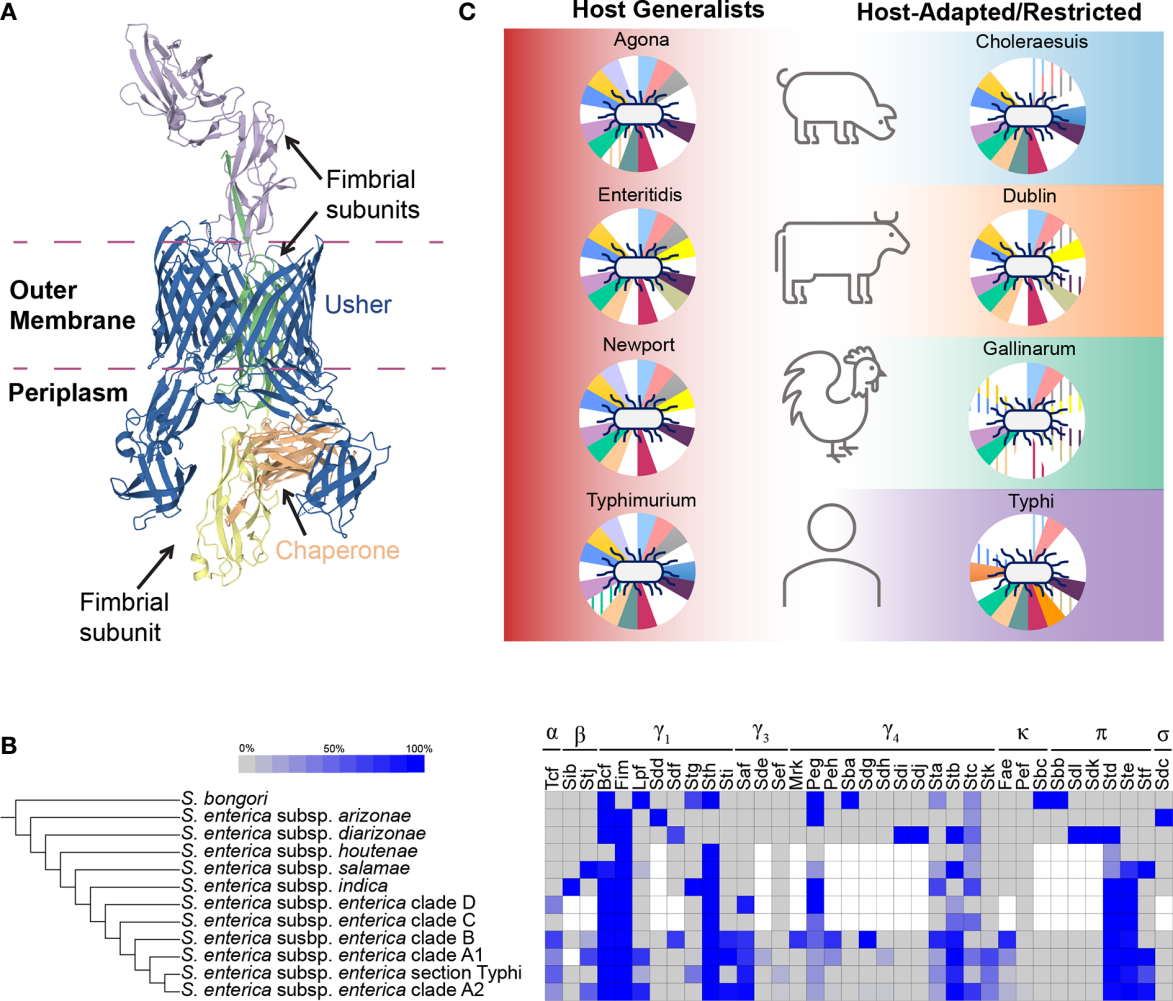
An overview of the *Salmonella* chaperone-usher fimbriome. **(A)** Structure of a chaperone-usher protein complex. The structure for Fim fimbria is shown as an example (PDB accession: 4J3O) (Geibel et al., 2013) to demonstrate the chaperone-usher biogenesis pathway. The membrane embedded usher (blue; FimD) accepts folded fimbrial subunits (FimF, FimG, and FimH) from the chaperone (orange; FimC) and translocates them across the outer membrane for elongation of the fimbria on the cell surface. **(B)** Overview of the distribution of known chaperone-usher fimbriae in *Salmonella*. Blue shading indicates the proportion of isolates in a given group that encoded the fimbrial gene cluster. White squares represent fimbrial gene clusters that were not included in analyses for a given *Salmonella* clade. The number of isolates included for each comparison varied: *S. bongori* (1–3 isolates), *S. enterica* subsp. *arizonae* and *diarizonae* (1–7 isolates), *S. enterica* subsp. *houtenae* (2–7 isolates), *S. enterica* subsp. *salamae* (2–9 isolates), *S. enterica* subsp. *indica* (1–3 isolates), and *S. enterica* subsp. *enterica* clades D (8 isolates), C (4 isolates), B (5–138 isolates), A1 (6–67 isolates), section Typhi (3–24 isolates), and A2 (10–186 isolates). For Tcf, Agf, Bcf, Fim, Lpf, Stg, Sth, Sti, Saf, Sef, Peg, Sta, Stb, Stc, Stk, Pef, Std, Ste, and Stf fimbriae, proportions of isolates in *S. enterica* subsp. *enterica* reflect only isolates from (Worley et al., 2018); for subsp. *arizonae*, *diarizonae*, *houtenae, salamae*, and *indica* datasets from multiple studies were compiled (Yue et al., 2012; Desai et al., 2013; Worley et al., 2018) as low numbers of isolates for these subspecies were reported. For *S. bongori*, data were compiled from (Fookes et al., 2011; Yue et al., 2012; Desai et al., 2013). Finally, for fimbriae Sdf, Sdg, Stj, Sdd/Smf, Sde, Mrk, Peh, Sba, Sdh, Sdi, Sdj, Fae, Sbc, Sbb, Sdk, Sdi, and Sdc, data were compiled from (Fookes et al., 2011; Yue et al., 2012; Desai et al., 2013). Fimbriae were considered as “present” if at least half of the genes in the operon were detected; pseudogenes were not considered in this analysis. In the Desai et al. (2013) dataset, Fae fimbria was referred to as “Skf” and Sdc fimbria was referred to as “Sas”. **(C)** A closer look at the distribution of chaperone-usher fimbriae among host-restricted (Gallinarum and Typhi), host-adapted (Choleraesuis and Dublin), and broad host range (Agona, Enteritidis, Newport, and Typhimurium) serovars. Background colors show associations with hosts (host generalists are shown in red to signify that they can infect all hosts shown, host-adapted/restricted serovars are aligned with the host that they are adapted/restricted to) (Hoelzer et al., 2011). Fimbriae are represented by the pie charts behind each *Salmonella*, with each slice of the chart representing an individual fimbria (clockwise from top light blue slice: Bcf, Fim, Lpf, Peg, Pef, Saf, Sef, Sta, Stb, Stc, Std, Ste, Stf, Stg, Sth, Sti, Stj, and Stk); slices appear colored in if the fimbria is present (i.e. white signifies absence of the fimbria) or have lines if **(i)** the fimbria is predicted to include hypothetically disrupted coding sequences for the gene encoding the usher protein or **(ii)** if more than half of the genes in the fimbrial gene cluster are predicted to be missing or are hypothetically disrupted coding sequences (Nuccio and Bäumler, 2014). Figure is based on data from (Nuccio and Bäumler, 2014) and reflects data for *S*. Agona SL483, *S*. Enteritidis P125109, *S*. Newport SL476, *S*. Typhi CT18, *S*. Typhimurium LT2, *S*. Gallinarum 287/91, *S*. Choleraesuis SC-B67, and *S*. Dublin CT_02021853.

Since the discovery of fimbriae in *Escherichia coli* in 1955, at least 39 different fimbriae have been described in *Salmonella* (Yue et al., 2012; Desai et al., 2013; Aviv et al., 2017; Cheng et al., 2019; Rehman et al., 2019). Fimbriae belong to four general categories depending on their morphology, function, or assembly pathway: (i) F pili, (ii) type IV fimbriae, (iii) curli, and (iv) chaperone-usher pathway fimbriae (reviewed in [Nuccio and Bäumler, 2007)]. Fimbriae of the chaperone-usher pathway represent the majority of fimbriae encoded by *Salmonella* (Nuccio and Bäumler, 2007; Rehman et al., 2019), and are therefore the focus of our discussion. Chaperone-usher pathway fimbriae, so named because these fimbriae utilize one or more chaperones to transfer folded fimbrial subunits to the usher protein (an integral outer membrane protein) for translocation across the outer membrane (**Figure 1A**), are categorized phylogenetically based on sequence conservation of the usher protein (Nuccio and Bäumler, 2007; Thanassi et al., 2007). The number of genes within a given chaperone-usher fimbrial gene cluster varies, but all include genes encoding (i) an usher protein, (ii) at least one chaperone, and (iii) a major fimbrial structural subunit; many fimbriae also include a minor fimbrial structural subunit known as a tip adhesin (Nuccio and Bäumler, 2007).

In 2007, Nuccio and Bäumler proposed the current classification scheme for chaperone-usher pathway fimbriae, which groups chaperone-usher fimbriae into three general categories based on the phylogenetic relatedness of the usher protein: (i) alternate, or α-fimbriae, (ii) classical, or β-, γ-, κ-, π-fimbriae; (iii) and archaic, or σ-fimbriae (Nuccio and Bäumler, 2007). Members of the γ-fimbriae are further sub-divided into classes γ_1_ through γ_4_ (Nuccio and Bäumler, 2007), although γ_2_ fimbriae have not been identified in *Salmonella* (Yue et al., 2012; Rehman et al., 2019). Among the 36 known chaperone-usher fimbriae produced by *Salmonella*, the alternative and archaic fimbriae include just one member each (Yue et al., 2012), and the remaining 34 represent members of the classical fimbriae with 23 belonging to the γ-fimbriae (**Figure 1B**) (Desai et al., 2013; Rehman et al., 2019). Additional novel fimbriae have since been proposed but the fimbrial gene cluster family for those was not reported (Desai et al., 2013; Aviv et al., 2017).

## Evolution of the *Salmonella* Chaperone-Usher Fimbriome: A Story of Gain and Loss

Recent advances in our understanding of the *Salmonella* chaperone-usher fimbriome have been catalyzed by the appreciable increase in the number of WGS data available. A synthesis of several studies (Fookes et al., 2011; Yue et al., 2012; Desai et al., 2013; Worley et al., 2018) examining the presence of fimbrial gene clusters among serovars belonging to the major lineages of *Salmonella* (Worley et al., 2018) suggests a complex history of multiple gain and loss events (**Figure 1B**).

### The Ancestral Chaperone-Usher Fimbriome: Fimbrial Gene Clusters Acquired Before the Divergence of *Salmonella* and *Escherichia*

At least seven *Salmonella* chaperone-usher fimbrial gene clusters are orthologous to fimbriae characterized in *Escherichia* (Nuccio and Bäumler, 2007), suggesting that these fimbrial gene clusters were most likely acquired prior to the divergence of these two genera approx. 140 million years ago (Yue et al., 2012; Desai et al., 2013). The Bcf fimbrial gene cluster [orthologue of Ycb in *E. coli* (De Masi et al., 2017)], originally named for its role in colonization of bovine hosts (Tsolis et al., 1999), is present in *S. bongori* and *S. enterica*, although this fimbrial gene cluster has been lost in subspecies *houtenae* (Desai et al., 2013). The conservation of a Fim (also called Type I fimbriae) orthologous fimbrial gene cluster [called Sfm (for *Salmonella* like fimbriae) among many lineages in *E. coli* (Wurpel et al., 2013)] supports that this fimbrial gene cluster was acquired prior to the divergence of *Salmonella* and *Escherichia* (Townsend et al., 2001), although this fimbrial gene cluster has since been lost in *S. bongori* (Fookes et al., 2011; Yue et al., 2012; Desai et al., 2013). Long polar fimbria [Lpf (Bäumler and Heffron, 1995)] shows a distinct evolutionary pattern characterized by an apparent acquisition from *E. coli* [also called Lpf in *E. coli* (Torres et al., 2002)] and conservation among *S. bongori* and some isolates of subspecies *salamae* and *enterica* (Worley et al., 2018); however some models based on a smaller set of isolates suggest the independent acquisition of the Lpf gene cluster by *S. bongori* and *S. enterica* subsp. *enterica* clade A serovars (Desai et al., 2013). The Peg fimbrial gene cluster [named for its original discovery in *S. enterica* subsp. *enterica* serovars Paratyphi A, Enteritidis, and Gallinarum (Thomson et al., 2008)] is detected broadly across both species and most *S. enterica* subspecies except for *diarizonae* and *houtenae. S. bongori* is predicted to have acquired the Sba fimbrial gene cluster from a most recent common ancestor (MRCA) shared with *E. coli*, although Sba has since been lost in *S. enterica* (Fookes et al., 2011; Yue et al., 2012). Finally, Sta [orthologue of *E. coli* Yad (Townsend et al., 2001; Wurpel et al., 2013)] and Stc [orthologue of *E. coli* Yeh (Townsend et al., 2001; Wurpel et al., 2013)] fimbrial gene clusters are found in *S. bongori* and *S. enterica* but show different distributions among the *S. enterica* subspecies (**Figure 1B**), suggesting different patterns of loss for these two fimbrial gene clusters (Yue et al., 2012; Desai et al., 2013).

### The Modern Chaperone-Usher Fimbriome: Fimbrial Gene Clusters (Likely) Acquired After Divergence From *Escherichia*

Following *Salmonella*’s divergence from *Escherichia*, the remaining chaperone-usher fimbrial gene clusters are hypothesized to have been acquired *via* horizontal gene transfer. While the exact mechanisms responsible for acquisition of the majority of *Salmonella* fimbrial gene clusters remain elusive, the presence of orthologous clusters in other Gammaproteobacteria (Nuccio and Bäumler, 2007; Desai et al., 2013; Stubenrauch et al., 2017), and the observation that multiple chaperone-usher fimbrial genes clusters are carried on plasmids [e.g., Pef and κ-fimbriae (Bäumler et al., 1996a; Nuccio and Bäumler, 2007; Aviv et al., 2017)] support their horizontal acquisition from other genera.

Although originally characterized in *S. enterica* subsp. *enterica* serovars Typhi [Stb, Std, Ste, Stf, Stg, and Sth (Townsend et al., 2001)] and Typhimurium [Stj (Mcclelland et al., 2001)], these fimbriae are distributed widely across multiple different *Salmonella* lineages (**Figure 1B**). The Stb and Std fimbrial gene clusters were likely acquired by subspecies *diarizonae* (Yue et al., 2012) and maintained in the majority of subspecies *enterica* isolates. The evolutionary history of Sdf, Ste, Stf, Stg, and Stj fimbrial gene clusters likely involved multiple acquisition events or a combination of acquisition and loss/retention by some subspecies (Yue et al., 2012; Desai et al., 2013). Last, the Sth fimbrial gene cluster is present in *S. bongori* and in most *S. enterica* subspecies (missing in subspecies *arizonae* and *diarizonae*); this fimbrial gene cluster was most likely acquired by *S. bongori* following its divergence from *Escherichia* (Desai et al., 2013) as this genomic region is missing from *E. coli* (Townsend et al., 2001) and no known *E. coli* fimbrial gene clusters with similar sequence homology have been identified (Nuccio and Bäumler, 2007).

The remaining *Salmonella* chaperone-usher fimbriae include those that have only been characterized in one species or subspecies. Multiple fimbrial gene clusters are found exclusively in non-subsp. *enterica* isolates: (i) Sbb and Sbc in *S. bongori* (Fookes et al., 2011; Yue et al., 2012), (ii) Sdd and Sdc, the only known σ-fimbria, in *S. enterica* subsp. *arizonae*, (iii) Sdi, Sdj, Sdl, and Sdk in *S. enterica* subsp. *diarizonae* (Yue et al., 2012), and (iv) Sib, a novel β-fimbria, in *S. enterica* subsp. *indica* (Desai et al., 2013). *S. enterica* subsp. *enterica* encodes 12 fimbriae that are not found in *S. bongori* or other *S. enterica* subspecies (**Figure 1B**). Tcf [for Typhi colonization factor (Folkesson et al., 1999)] and Saf are present in isolates representing all currently known subsp. *enterica* clades except for clade C. Sti, Peh, Sdg, and Sdh fimbrial gene clusters are found in multiple subsp. *enterica* clades, but are largely missing from section Typhi isolates (Sti is present in one serovar, Kintambo, in section Typhi), suggesting the selective loss of these fimbriae in section Typhi isolates. Conversely, Stk and Fae fimbrial gene clusters are found in some isolates representing subsp. *enterica* clades B, A1, Typhi, and A2. The plasmid-encoded Pef fimbrial gene cluster was only detected in a handful of serovars including Nottingham (clade B) and four serovars in clade A2 that had previously been shown to carry the *Salmonella* virulence plasmid (i.e., serovars Choleraesuis, Enteritidis, Bovismorbificans, and Typhimurium) (Cheng et al., 2019). The Sef [*Salmonella* Enteritidis fimbriae (Clouthier et al., 1993)] fimbrial gene cluster is found in multiple clade A2 serovars as well as in some section Typhi isolates (Yue et al., 2012). Finally, the Sde and Mrk [Mannose resistant *Klebsiella*-like fimbriae (Wilksch et al., 2011)] fimbrial gene clusters have only been reported in serovars Tennessee (clade A1) and Montevideo (clade B), respectively (Yue et al., 2012).

With a more complete picture of the *Salmonella* chaperone-usher fimbriome, several key themes arise. First, many chaperone-usher fimbrial gene clusters are detected broadly across both *S. bongori* and *S. enterica*, suggesting the early acquisition of these chaperone-usher fimbrial gene clusters. Second, very few chaperone-usher fimbrial gene clusters are serovar-specific. While this may be a reflection of the specific bioinformatic approaches used (i.e., identity cut-off used) or the isolates in the comparison, it suggests that while fimbriae may contribute to host/niche adaptation, they are likely only a part of the process as many other factors also play a role (e.g., changes in metabolic pathways (Nuccio and Bäumler, 2014), immunological naivety of the host (Bäumler et al., 2000), etc.). Last, the currently available data highlight multiple “rare” fimbrial gene clusters, where future characterizations including a broader range of isolates will be necessary to extend our knowledge of the evolutionary events associated with these fimbriae.

## Making an Entrance—Examining a Role for Chaperon-Usher Fimbriae in Mediating Interactions Within and Between Hosts

As reviewed previously (Rivera-Chavez and Bäumler, 2015), *Salmonella* is ill-suited to compete with the resident anaerobic microbiota within the lumen of the gut (Tsolis and Bäumler, 2020). Therefore, *Salmonella* uses different strategies upon entering the gut to either escape to a slightly less competitive environment or modify the environment to one that favors its expansion (Tsolis and Bäumler, 2020). Fimbriae are proposed to play a key role in mediating initial interactions with host cells (Fàbrega and Vila, 2013). The majority of the host cell surface receptors that are recognized and bound by fimbriae remain elusive, although several surface glycans have been identified as facilitating adhesion (**Table 1**): Std (π-fimbriae) binds to terminal α-1,2 fucose (Chessa et al., 2009; Suwandi et al., 2019), Fim (γ_1_-fimbriae) binds to mannose (Duguid et al., 1966), and Pef (κ-fimbriae) binds Galβ1-4(Fucα1-3)GlcNAc [also called the Le^x^ histo-blood group antigen (Chessa et al., 2008)]. These observations suggest that fimbriae may mediate preferential binding to different glycans, thereby facilitating *Salmonella’s* ability to bind to different host cell surfaces either within the same host (tissue tropism) or in different hosts (host tropism).

**Table 1:** Summary of fimbriae with known host receptors.

Chaperone-usher clade	Fimbria	Host cell receptor	Cell types bound^1^	References
γ_1_	Fim	Mannose residues on glycoprotein 2	M cells	(Duguid et al., 1966; Hase et al., 2009)
γ_1_	Lpf	Unknown	M-like cells	(Bäumler et al., 1996b)
γ_1_	Stg	Unknown	Enterocytes	(Gonzales et al., 2017)
κ	Pef	Galβ1-4(Fucα1-3)GlcNAc	Unknown	(Chessa et al., 2008)
π	Std	α-1,2 fucose	Colonic and cecal intestinal epithelial cells	(Chessa et al., 2009; Suwandi et al., 2019)

^1^Types of host cells predicted to be the preferential binding target of the fimbria.

### A Role for Chaperone-Usher Fimbriae in Tissue Tropism?

*Salmonella* strains encode an average of 5–14 different fimbrial gene clusters (McClelland et al., 2001; Townsend et al., 2001; Yue et al., 2012; Worley et al., 2018) with multiple fimbriae being co-expressed *in vivo* (Humphries et al., 2003; Laniewski et al., 2017). During an infection, nontyphoidal *Salmonella* (e.g., *S*. Typhimurium) capitalize on a pro-inflammatory response to generate host-derived nitrate released by immune cells (Rivera-Chavez and Bäumler, 2015) to favor their expansion in the gut lumen. In contrast, *S*. Typhi favors an anti-inflammatory approach by evading (Winter et al., 2015) immune surveillance and colonizing extraintestinal sites such as the gallbladder (Gunn et al., 2014). Reflective of these strategies, different fimbriae are proposed to facilitate nontyphoidal *Salmonella*’s preferential binding to M cells (Hase et al., 2009) and typhoidal *Salmonella*’s preferential binding to enterocytes (Gonzales et al., 2017). *S*. Typhimurium FimH, the tip adhesin of Fim, binds glycoprotein 2 (GP2) on M cells in a mannose-dependent manner (Hase et al., 2009), while Lpf facilitates binding to M-like cells (Gonzales et al., 2017) and murine Peyer’s patches [**Table 1** (Bäumler et al., 1996b)]. Conversely, Stg fimbriae in *S*. Typhi are thought to promote binding to enterocytes (Gonzales et al., 2017), although in at least some *S*. Typhi the Stg usher protein is likely non-functional due to frame shift mutations leading to pseudogene formation (Townsend et al., 2001; Dufresne et al., 2018).

After breaching the gut epithelium, *S*. Typhimurium uses Sti, Saf, Agf (curli, non-chaperone-usher fimbriae), and Stc fimbriae at extraintestinal sites, as quadruple deletion mutants show a reduced colonization of the spleen and liver compared to wild type strains (Laniewski et al., 2017). *S*. Typhi Agf has also been shown to enable binding to the gallbladder epithelium (Gonzalez-Escobedo and Gunn, 2013), supporting a role for this fimbria in promoting chronic colonization of *S*. Typhi in the gallbladder (Gonzalez-Escobedo et al., 2011). Several fimbriae have been implicated in facilitating long-term intestinal colonization of 129X1/SvJ [Saf, Bcf, Sti, and Std (Lawley et al., 2006)] and CBA mice [Lpf, Bcf, Stb, Stc, Std, and Sth (Weening et al., 2005)]; as deletion of these fimbriae did not result in a reduced recovery of *S*. Typhimurium from fecal samples of BALB/c mice at five days post infection (Weening et al., 2005), it appears that these fimbriae instead effect colonization in a temporal-manner, at different stages over the course of an infection. Finally, multiple studies have shown that chaperone-usher fimbriae undergo phase variation, a process in which expression of fimbriae in the bacterial population is heterogeneous (Humphries et al., 2001; Humphries et al., 2005; Kolenda et al., 2019). Together, these observations support a role for multiple chaperone-usher fimbriae in facilitating tissue tropism in both acute and chronic infections for both typhoidal and nontyphoidal *Salmonella*, but also suggest redundant roles for many fimbriae as deletion/inactivation of a single fimbrial gene cluster often has negligible effects on virulence *in vivo*.

### It Is Complicated—A Combination of Fimbrial Adhesin Allelic Diversity, Pseudogene Formation, and Differential Fimbrial Expression Support a Role for Chaperone-Usher Fimbriae in Host Tropism

Together with the observation that many chaperone-usher fimbriae are distributed across multiple lineages (and serovars), several studies have suggested that the mere presence of fimbrial gene clusters cannot fully explain the observed patterns of host tropism (Chaudhuri et al., 2013; Zhang et al., 2019), highlighting the complexity of the relationship between fimbriae and virulence (Humphries et al., 2001). One possible explanation is that analyses that only consider presence/absence data do not account for pseudogene formation among fimbrial genes, which has been demonstrated previously for multiple host-adapted/restricted serovars Typhi (Townsend et al., 2001), Dublin (Nuccio and Bäumler, 2014; Langridge et al., 2015), Gallinarum (Clayton et al., 2008), Choleraesuis and Paratyphi C (Nuccio and Bäumler, 2014) (**Figure 1C**). Another possibility is that the conformation of the tip adhesin mediates binding to different host cells, as several studies have documented that different alleles of fimbrial adhesins are associated with isolation from different hosts (Kisiela et al., 2012; Yue et al., 2015; De Masi et al., 2017). Furthermore, there is some evidence to suggest that these adhesins may also use different receptors when infecting different hosts (Grzymajło et al., 2013; Grzymajlo et al., 2017). Several studies have suggested that different conformations of the tip adhesins of Fim (Kisiela et al., 2012; Yue et al., 2015; De Masi et al., 2017), and Bcf and Stf (De Masi et al., 2017) fimbriae among nontyphoidal serovars enhance preferential binding to different host cell lines. For example, the allelic variation in *fimH* alleles from host-adapted/host-restricted serovars (e.g., Dublin and cattle, Typhi and humans, etc.) was associated with preferential binding to cell lines representing the host that the serovar was isolated from (e.g., Dublin and bovine cells) (Yue et al., 2015), corroborating the results of previous studies that suggested that different *fimH* alleles allowed for selective binding to different host cell types (Boddicker et al., 2002; Kisiela et al., 2012; Grzymajło et al., 2013). Last, fimbrial gene expression is a complex process that involves regulators encoded in the fimbrial gene cluster that directly regulate transcription of genes in the cluster (Kolenda et al., 2019), as well as global regulators including H-NS (Hurtado-Escobar et al., 2019), CsrA (Sterzenbach et al., 2013), and HdfR (García-Pastor et al., 2019). These complex regulatory pathways may also partially explain why strains representing different serovars show variable expression of fimbriae, and why deletion of these chaperone-usher fimbrial gene clusters in has varying effects on influencing virulence (Azriel et al., 2017).

Overall, these studies suggest that fimbriae-mediated adhesion to host cells is a complex process. Therefore, bigger picture assessments of the roles that these fimbriae play in mediating host interactions will require both an attention to detail (i.e., specific sequence of adhesins, distribution of receptors in different hosts) as well as considerations for the role of phase variation in facilitating evasion of the host immune system (Humphries et al., 2001).

## Missing Pieces of the *Salmonella* Chaperone-Usher Fimbriome Puzzle

The appreciable diversity of *Salmonella* serovars is mirrored by its diverse chaperone-usher fimbriome. While some fimbriae, such as Fim and Agf, have been studied extensively due in part to their expression under certain standard lab culturing conditions (Laniewski et al., 2017; Kolenda et al., 2019), discovery of novel fimbrial gene clusters continues with the expanded use of whole genome sequencing in public health and research efforts. With current initiatives aimed at utilizing genomic data for source prediction to facilitate foodborne outbreak investigations (Zhang et al., 2019; Merlotti et al., 2020), an enhanced understanding of the role that diverse chaperone-usher fimbriae, and potentially different conformations of their adhesins, play in the colonization of a wide range of hosts is warranted. Future investigations to understand the sequence diversity and conservation of chaperone-usher fimbriae among lesser studied *Salmonella* subspecies and serovars will be important for determining patterns of chaperone-usher fimbriae associated with different hosts. Furthermore, structural data of fimbrial components that facilitate interactions with host receptors will require the development and characterization of additional cell culture/organoids and animal models to expand our understanding of the role of chaperone-usher fimbriae in a broader range of tissues and hosts. These, combined with more basic inquiries to understand transcriptional and translational regulatory mechanisms to identify which host signals govern expression of different fimbriae, represent important missing pieces in our understanding of the *Salmonella* chaperone-usher fimbriome puzzle.

## Author Contributions

This review article was conceptualized by RC and MW. RC and MW wrote, reviewed, and edited the original and final drafts. Funding was secured by RC. All authors contributed to the article and approved the submitted version.

## Funding

Funding for this review was provided by USDA 2020-67034-31905 awarded to RC.

## Conflict of Interest

The authors declare that the research was conducted in the absence of any commercial or financial relationships that could be construed as a potential conflict of interest.
